# Chemical Behavior and Bioactive Properties of Spinorphin Conjugated to 5,5′-Dimethyl- and 5,5′-Diphenylhydantoin Analogs

**DOI:** 10.3390/ph17060770

**Published:** 2024-06-12

**Authors:** Stela Georgieva, Petar Todorov, Jana Tchekalarova, Subaer Subaer, Petia Peneva, Kalin Chakarov, Hartati Hartati, Sitti Faika

**Affiliations:** 1 Department of Analytical Chemistry, University of Chemical Technology and Metallurgy, 1756 Sofia, Bulgaria; 2 Department of Organic Chemistry, University of Chemical Technology and Metallurgy, 1756 Sofia, Bulgaria; pepi_37@abv.bg (P.T.);; 3 Institute of Neurobiology, Bulgarian Academy of Sciences, 1113 Sofia, Bulgaria; 4 Material Physics Laboratory, Physics Department, Universitas Negeri Makassar (UNM), Makassar 90223, Indonesia

**Keywords:** spinorphin, peptide, electrochemical characterization, bioactive properties, spectral properties, acid-based constant

## Abstract

The discovery of new peptides and their derivatives is an outcome of ongoing efforts to identify a peptide with significant biological activity for effective usage as a possible therapeutic agent. Spinorphin peptides have been documented to exhibit numerous applications and features. In this study, biologically active peptide derivatives based on novel peptide analogues of spinorphin conjugated with 5,5′-dimethyl (Dm) and 5,5′-diphenyl (Ph) hydantoin derivatives have been successfully synthesized and characterized. Scanning electron microscopy (SEM) and spectral methods such as UV-Vis, FT-IR (Fourier Transform Infrared Spectroscopy), CD (Circular Dichroism), and fluorimetry were used to characterize the microstructure of the resulting compounds. The results revealed changes in peptide morphology as a result of the restructuring of the aminoacidic sequences and aromatic bonds, which is related to the formation of intermolecular hydrogen bonds between tyrosyl groups and the hydantoin moiety. Electrochemical and fluorescence approaches were used to determine some physicochemical parameters related to the biological behavior of the compounds. The biological properties of the spinorphin derivatives were evaluated in vivo for anticonvulsant activity against the psychomotor seizures at different doses of the studied peptides. Both spinorphin analog peptides with Ph and Dm groups showed activity against all three phases of the seizure in the intravenous Pentylenetetrazole Seizure (ivPTZ) test. This suggests that hydantoin residues do not play a crucial role in the structure of spinorphin compounds and in determining the potency to raise the seizure threshold. On the other hand, analogs with a phenytoin residue are active against the drug-resistant epilepsy test (6-Hz test). In addition, bioactivity analyses revealed that the new peptide analogues have the potential to be used as antimicrobial and antioxidant compounds. These findings suggest promising avenues for further research that may lead to the development of alternative medicines or applications in various fields beyond epilepsy treatment.

## 1. Introduction

Bioactive peptides include a wide range of peptides, from simple dipeptides to complex linear and cyclic structures [1,2]. Much research has been performed on the bioactivity of peptides and how they can help reduce chronic diseases [3,4]. These studies have looked at their antibacterial, anti-inflammatory, anticancer, and antidiabetic effects [3,4]. It is possible to extract antimicrobial peptides from natural materials, but there are obstacles such as limited production and unexpected contaminants that make the process difficult. On the other hand, the search for new peptides that can be used for different purposes, such as anticonvulsant agents or morphine substitution analgesics, is mainly aimed at developing peptides or peptidomimetic analogues that have higher biological stability and receptor selectivity [5,6,7]. As a result, peptide synthesis is increasingly used in various scientific investigations. Peptide design allows the modification of amino acid residues to enhance their antibacterial and immunomodulatory properties, and even to improve their resistance to degradation. An advantage of the large-scale production of synthetic peptides for industry is their ability to be expressed in other organisms using heterologous expression techniques [8]. On the other hand, as mentioned in our previous paper, spinorphin is a heptapeptide consisting of the amino acid sequence Leu-Val-Val-Tyr-Pro-Trp-Thr. It is classified as an endogenous, nonclassical opioid peptide belonging to the hemorphin family (LVV-hemorphin-4) [9,10]. Spinorphin was first isolated from the spinal cord of a cow [11], and its sequence corresponds to a part of the hemoglobin molecule. The compound is analgesic and inhibits the enkephaline-degrading enzyme [12]. Spinorphin has recently been shown to inhibit neutrophil activation generated by the chemotaxic N-formyl peptide N-formylmethionylleucylphenylalanine (fMLF), indicating a potential role as a negative regulator of endogenous inflammation [10]. Compared to pain pathways that are unaffected by morphine, spinorphin has pain-relieving properties [13,14,15] and significantly reduces the effects of bradykinin (BK) on nociceptive flexor responses [16]. As can be seen, the spinorphin molecule has shown numerous physiological effects such as antinociceptive, antiallodynic, and anti-inflammatory properties [17]. When a spinorphin molecule is mixed with structures that have been shown to have a biological effect, they work together to improve biological functions [9]. Phenytoin is a hydantoin-based anticonvulsant drug commonly used to inhibit the spread of seizures in the brain by reducing the excessive activation of neurons [18]. Research has shown that the compound affects the movement of substances across cell membranes, reducing the amount of sodium ions within the cell and causing hyperpolarization of the cell membrane. Immediately after entering the brain, serotonin and noradrenaline levels increase, while acetylcholine levels decrease. Recent research suggests that phenytoin has neuroprotective effects, protecting nerves from damage and possibly slowing the progression of damage [19]. Changing the hydantoin molecular structure or combining it with other molecular sequences can make the basic biological effects of phenytoin stronger or weaker [20,21]. Similar conclusions were made when it was bound to a peptide molecule as it was noted to transition from a neutral role to an enhancing role regarding the biological activities of the peptide depending on its amino acid sequences [9]. In this regard, the aim of this study was to explore the potential for enhancing the bioactivity of spinorphin molecules conjugated with hydantoin derivatives by investigating their microstructure and bioactivity, including antimicrobial, antioxidant, and anticonvulsant properties. The correlation between the microstructure and the properties of the synthetic peptides was studied by applying various techniques, including voltamperometry (VA), UV-Vis spectroscopy, FTIR spectroscopy (Fourier Transform Infrared Spectroscopy), circular dichroism (CD), and scanning electron microscopy (SEM).

## 2. Results and Discussion

### 2.1. Chemistry of Spinorphine Derivatives

**Scheme 1:**
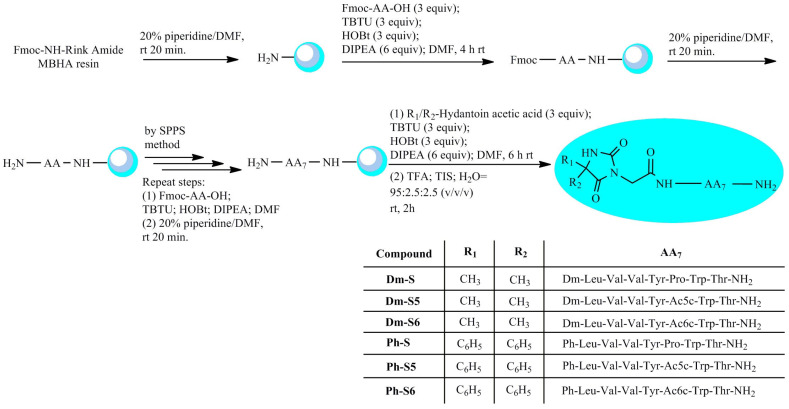
Schematic pathway of SPPS–Fmoc strategy in synthesizing new 5,5′-dimethyl- and 5,5′-diphenylhydantoin-conjugated spinorphin derivatives.

Six novel 5,5′-dimethyl- and 5,5′-diphenylhydantoin-conjugated spinorphin analogs designed as potential anticonvulsant agents (Dm-S, Dm-S5, Dm-S6, Ph-S, Ph-S5, and Ph-S6) were synthesized by a solid-phase peptide synthesis (SPPS)-Fmoc (9-fluorenylmethoxy-carbonyl) strategy using TBTU (2-(1H-benzo-triazole-1-yl)-1,1,3,3-tetramethyl-uronium tetrafluoro-borate), an efficient peptide-coupling reagent (Scheme 1). The main goal of the present study was to design, synthesize, and evaluate new N-modified hybrid analogs of spinorphins containing hydantoin residues that possess stronger anticonvulsant activity by incorporating C-5 substituted hydantoins. We focused on spinorphin, which has been reported to have the most biological activity [10,11,14,16,17].

Since the amino acid proline is known to be essential for biological effects, the structure–activity relationship was investigated by replacing natural proline with unnatural steric-restricted amino acids such as 1-aminocyclopentanecarboxylic acid (Ac5c) and 1-aminocyclohexane carboxylic acid (Ac6c) (peptides: Dm-S5, Dm-S6, Ph-S5, and Ph-S6). Conformationally restricted amino acids, such as Ac5c and Ac6c, are increasingly used in medicinal chemistry because they retain the conformation appropriate for biological action [9,22,23]. This allowed us to evaluate the influence of the peptide fragment, native amino acid Pro and non-natural amino acids, and peptide N-terminal alterations with C-5 substituted dimethyl/diphenylhydantoin derivatives. To obtain the final N-modified spinorphins (Dm-S, Dm-S5, Dm-S6, Ph-S, Ph-S5, and Ph-S6), it is first necessary to synthesize the corresponding modified 5,5′-dimethyl/diphenylhydantoin acetic acids. They were synthesized using the approach that we disclosed in [18].

**Figure 1:**
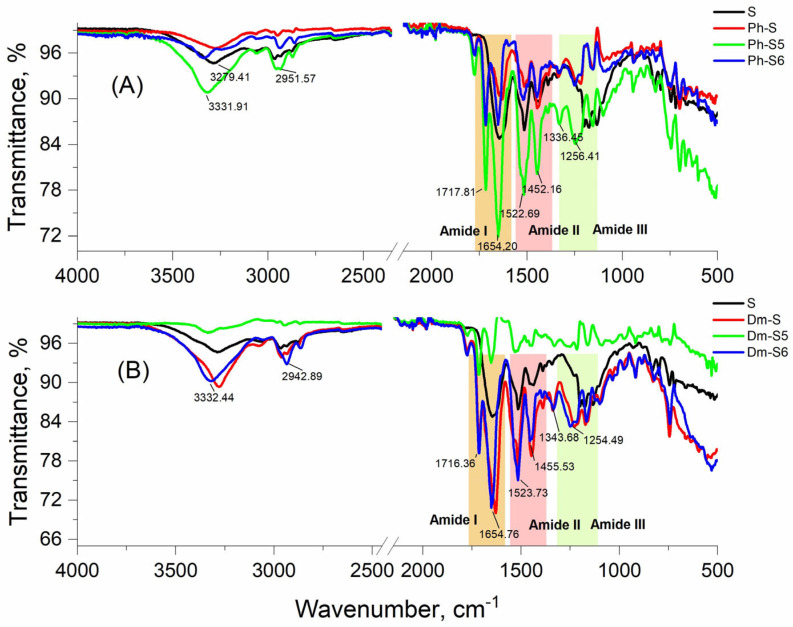
FTIR spectrum of peptides S and its analogous: (**A**) Ph-S, Ph-S5, and Ph-S6 and (**B**) Dm-S, Dm-S5, and Dm-S6 showing the bands of amides I, II, and III.

**Figure 2:**
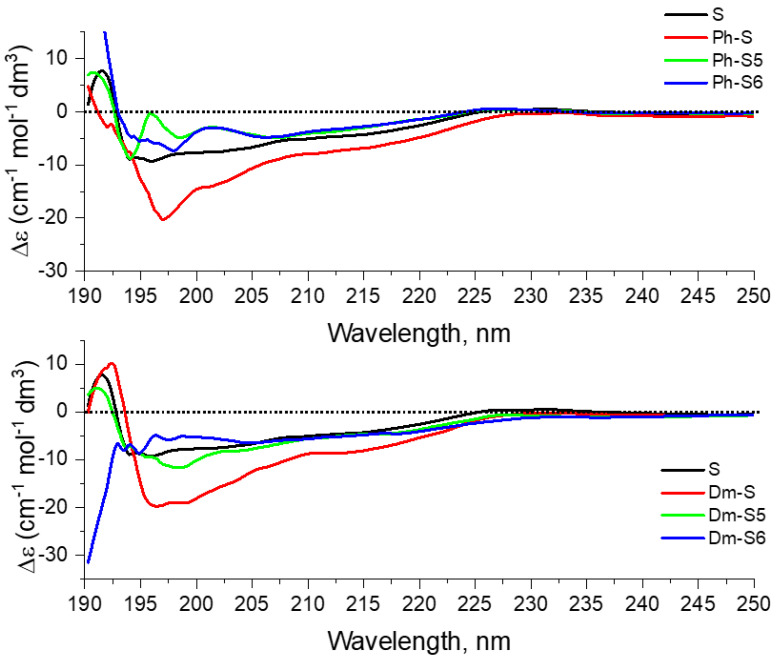
CD spectra between 190 and 250 nm of the studied hydantoin ring-conjugated peptide derivatives.

IR spectra were recorded for the compounds under study to illustrate the characteristic groups associated with their structure and conformational state. The amide I and amide II bands are the main signals in the infrared spectrum of the peptides and are sensitive to the conformation of these types of compounds. The Amide II band is mainly due to the bending vibration of the N—H bond and the stretching vibration of the C—N bond [24]. Significant bands related to different peptide conformations were also identified in the spectral range below 1400 cm^−1^, namely in the amide III region. Typical peak combinations are as follows: 1655 cm^−1^ with bands at 1175, 1305, 2950, and 3330 cm^−1^ corresponding to an alpha-peptide structure. The peptides studied have bands corresponding to non-aggregated molecules and possess both an α-helix and a β-sheet. The strong absorption of the compounds at 1653 cm^−1^ is attributed to the presence of solid-state α-helical conformers (Figure 1). However, this absorption lacks the corresponding band in the amide II region at 1545 cm^−1^, which is often indicative of this specific type of structure. The observation line is located at a wavelength number of 1523 cm^−1^, indicating the presence of a β-sheet structure. In addition, the presence of beta structures is indicated by peaks at 1254 cm^−1^ [24]. The band observed at 1770 cm^−1^ could also be related to β-sheets. In the amide III region, bands of 1336 cm^−1^ and 1254 cm^−1^ confirm the presence of β-turn conformers. The infrared absorption of Ph-S, Ph-S6, and Dm-S5 shows a significant decrease in the absorption maximum compared to the reference spinorphin (S). These results are likely due to the significant amounts and dimensions of porosity present on the surface of the peptides under investigation, as revealed by the SEM examination (see the following section below). On the other hand, the presence of certain amino acids is more or less likely to be found in α-spirals or β-sheets. For example, the presence of the amino acid proline in compounds S, Dm-S, and Ph-S prevents the formation of a peptide helix by attaching to adjacent amino acids in the peptide backbone. This prevents the chain from folding and is normally incompatible with helix formation. Similarly, the presence of amino acids such as tryptophan, tyrosine, and phenylalanine, which have large ring-shaped structures, are often found in β-folded sheets, likely because the structure of the β-folded leaf provides ample room for side chains. To confirm the conformational state of the peptide derivatives, their spectra were recorded when interacting with circularly polarized light (Figure 2). Conventional spectroscopy (Circular Dichroism, CD) was used to evaluate the interaction in the near-UV region. It can be seen that different intensity bands are obtained depending on the type of compound. It can be seen from the figure that the proline-containing peptide derivatives have a high content of conformational isomers of the β-sheet and a lower content of β-turns, as they show large absorption bands in the negative region at ~197 nm. The remaining peptides with substituted proline have a low structural level due to their high content of non-chiral Ac5c and Ac6c (Figure 2). Therefore, both negative and positive signals in the CD spectra of both peptides are very low. The presence of bands in the spectra of Dm-S5, Dm-S6, Ph-S5, and Ph-S6 at 190 nm, as well as the shoulder at 205 nm, indicates a significant number of α-helical conformers in the aqueous solutions of these four peptides. With regard to pure spinorphin, it is likely that it has a beta-sheet structure since its spectrum shows similarity to the proline-containing compounds, but with less pronounced peaks due to the lower number of chiral atoms.

**Figure 3:**
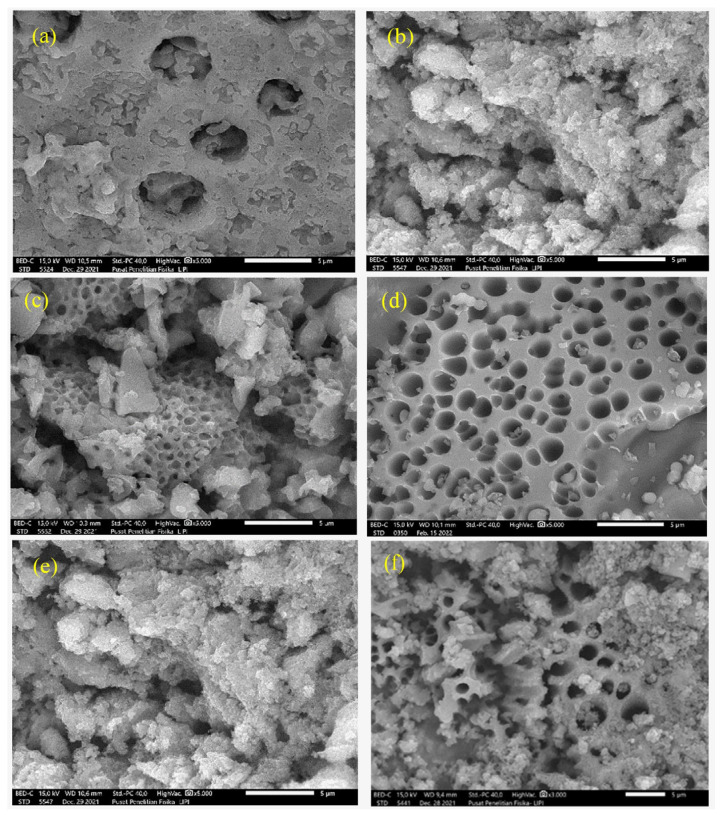
SEM images at a scale bar of 5 µm of (**a**) S, (**b**) Dm-S, (**c**) Dm-S5, (**d**) Dm-S6, (**e**) Ph-S, and (**f**) Ph-S5.

The morphology of the new peptide molecules was studied in order to show their microstructure, which is important to demonstrate the integrity of the molecule without its transition to an aggregated state to which peptide compounds are very sensitive. The spinorphin image (S) in Figure 3a shows the pore phase as well as the spinorphin granules, which are flat and dense. The morphology of the peptide analogues in Figure 3b shows significant changes in the density of the derivatives compared to the reference due to the addition of more carbon and hydrogen atoms and the probable formation of intermolecular hydrogen bonds between tyrosyl groups and hydantoin residues. The formation of more uniformly distributed pores, especially in the Dm-S6 samples, corresponds to the morphology of the peptides reported by Hincapié et al, who studied polycationic antimicrobial peptides active against Pseudomonas aeruginosa and Staphylococcus aureus [25].

### 2.2. Analytical Characteristics

**Figure 4:**
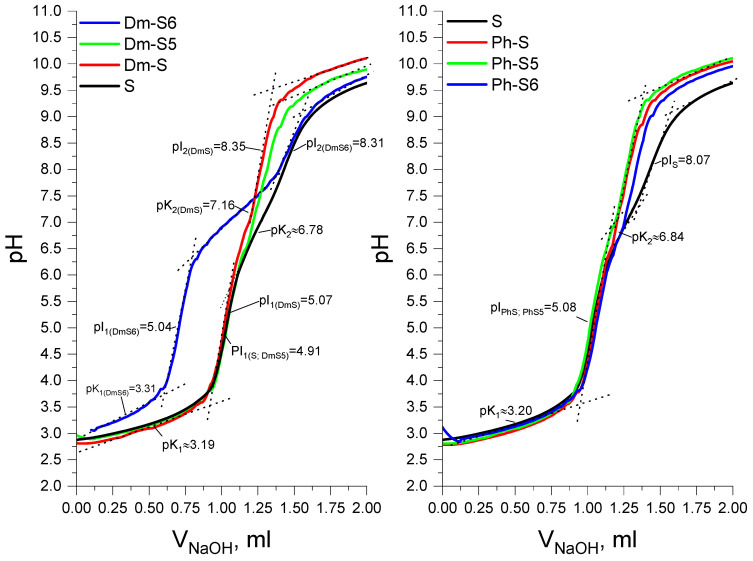
Plot of pH vs. V_NaOH_ of 12 µmol hybrid peptides. Dashed lines are tangent to corresponding sections of titration curves to define corresponding distances relevant to determination of isoelectric points and acid-base constants.

The acid–base properties of the compounds were evaluated in an aqueous environment to analyze their structural behavior in a physiological-like setting. Figure 4 shows the titration curves for all compounds. The values of the acid constants and those of their isoelectric points of existence as zwitterions are given in Table 1. The new peptide molecules are weak protoliths with almost identical values of the constants. The obtained values correspond to peptide structures that are coupled to hydantoin derivatives and are similar in nature [18]. Thus, it can be concluded that the conjugated part of the molecule does not significantly affect its acidic properties. All compounds exhibit an ionic form at physiological pH, and in environments of pH~5 and 8.30, they are in insoluble form and unstable for biological activity, respectively (Table 1).

**Figure 5:**
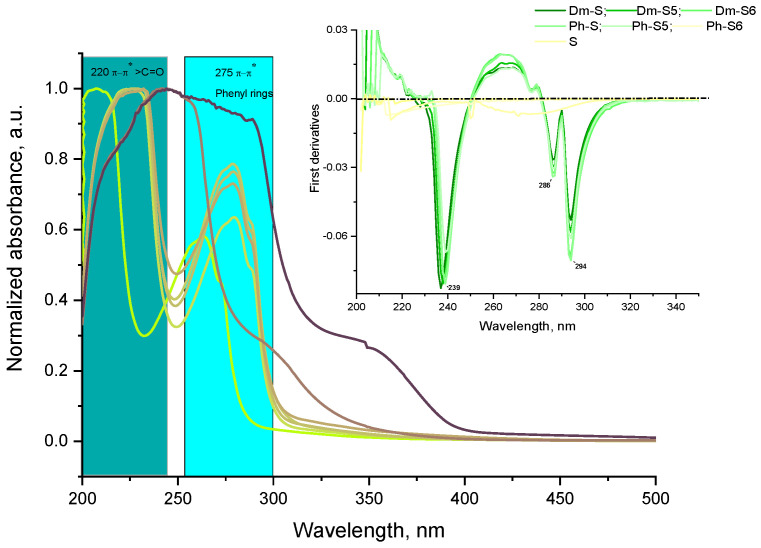
UV-Vis spectra of solutions of peptide derivatives at concentrations: S (C_S_ = 1.584 × 10^−3^ mol L^−1^); Dm-S (C_Dm-S_ = 1.353 × 10^−3^ mol L^−1^ ); Dm-S5 (C_Dm-S5_ = 1.335 × 10^−3^ mol L^−1^); Dm-S6 (C_Dm-S6_ = 1.387 × 10^−3^ mol L^−1^); Ph-S (C_Ph-S_ = 1.230 × 10^−3^ mol L^−1^); Ph-S5 (C_Ph-S5_ = 1.469 × 10^−3^ mol L^−1^); Ph-S6 (C_Ph-S6_ = 1.160 × 10^−3^ mol L^−1^); inserted graphic represents the first-derivative spectra of the same peptides.

**Figure 6:**
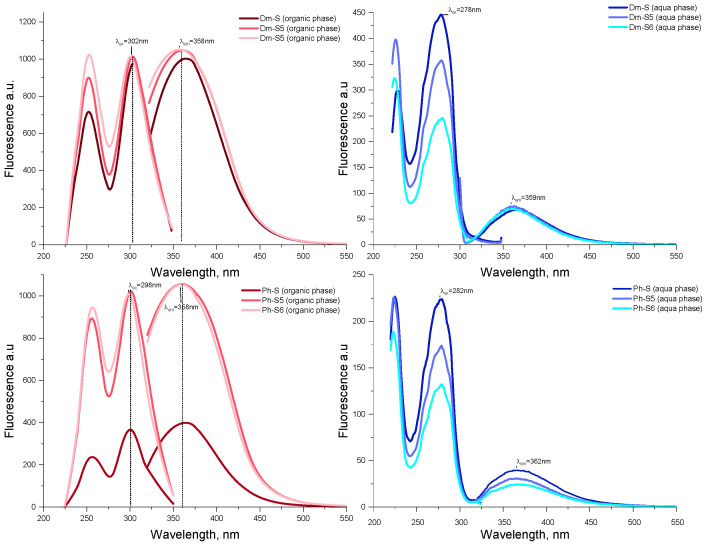
Fluorescence spectra of the studied peptide compounds at different concentrations obtained after distribution in two phases: organic: 1-octanol and aqua: phosphate buffer (NaH_2_PO_4_/Na_2_HPO_4_, pH 7.41 ± 0.01).

Spinorphin derivatives exhibit physicochemical properties similar to those of molecules containing chromophoric groups in the UV-Vis spectra. Regardless of the substituents on the hydantoin nucleus, all compounds show the same spectral relationships (Figure 5). The presence of peptide bonds is indicated by the high-intensity bands at 200–230 nm [26]. The peptide bond in the reference sample (pure spinorphin, S) is centered at 232 nm and shifts to a shorter wavelength in the remaining derivatives. The presence of two benzyl parts in the phenytoin leads to a hyperchromic effect, expressed in an increase in the intensity of the spectral band in the aromatic region 280–310 nm, compared to the 5,5′-dimethyl derivatives. Similarly, the aromatic region is centered at 280 nm in all samples, but the absorption increases significantly for the reference sample, indicating that the hydantoin substituents involved in conjugation with the peptide chain reduce the absorption of tyrosine and tryptophan, with the amino acids possessing spectral absorption maxima (Figure 5). The wavelength at which the compounds were irradiated was identified from the absorption spectra, allowing the molecule to be excited and the energy of the emitted radiation to be measured. The resulting fluorimetries spectra (Figure 6) were used to calculate the partition coefficient (LogP) of the compounds between two phases: organic and aqueous, with the value indicating the compounds’ lipophilicity and hydrophobicity. The value of LogP is often used to determine whether a substance with pharmacological or biological activity possesses properties that make it active after consumption according to “Lipinsky’s Rule of Five” technique, which uses logP as the basic criterion, with a value lower than 5 [27]. For the peptide modifications studied by us, it can be seen that the solutions of the compounds in the two media give different emission intensities, which can be attributed both to the different sensitivities of the compounds in the two media and the different concentrations resulting from the distribution carried out. Therefore, the intensities of the compounds in both phases, organic and inorganic, were first measured, and then standard stock solutions were added to find the coefficient of the analytical function relative to the two media. The calculated values of the distribution coefficients are given in Table 1. We can conclude that all compounds have approximately the same positive values of the distribution coefficients and are more likely to be lipophilic than hydrophilic. This indicates that the unions tend to accumulate in adipose tissue, pass through the lipid layers of biological membranes, and therefore have the potential for bioaccumulation in organisms.

### 2.3. Determination of Hydrolytic Stability

**Figure 7:**
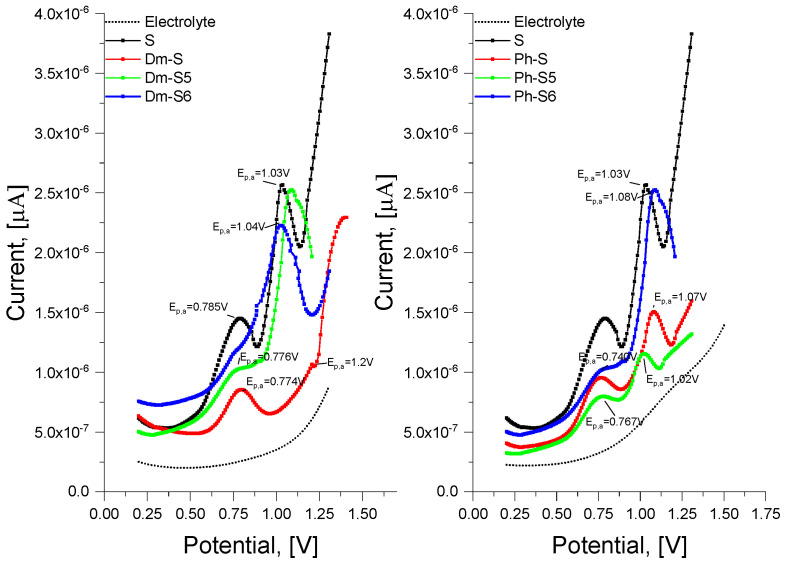
DPP voltamperograms of the test compounds in phosphate buffer solution and concentrations: S (C_S_ = 1.584 × 10^−5^ mol L^−1^); Dm-S (C_Dm-S_ = 1.353 × 10^−5^ mol L^−1^ ); Dm-S5(C_Dm-S5_ = 1.335 × 10^−5^ mol L^−1^); Dm-S6 (C_Dm-S6_ = 1.387 × 10^−5^mol L^−1^); Ph-S (C _Ph-S_ = 1.230 × 10^−5^ mol L^−1^); Ph-S5 (C_Ph-S5_ = 1.469 × 10^−5^ mol L^−1^); Ph-S6(C _Ph-S6_ = 1.160 × 10^−5^ mol L^−1^); GC electrode as working electrode, Ag/AgCl as a reference electrode.

**Figure 8:**
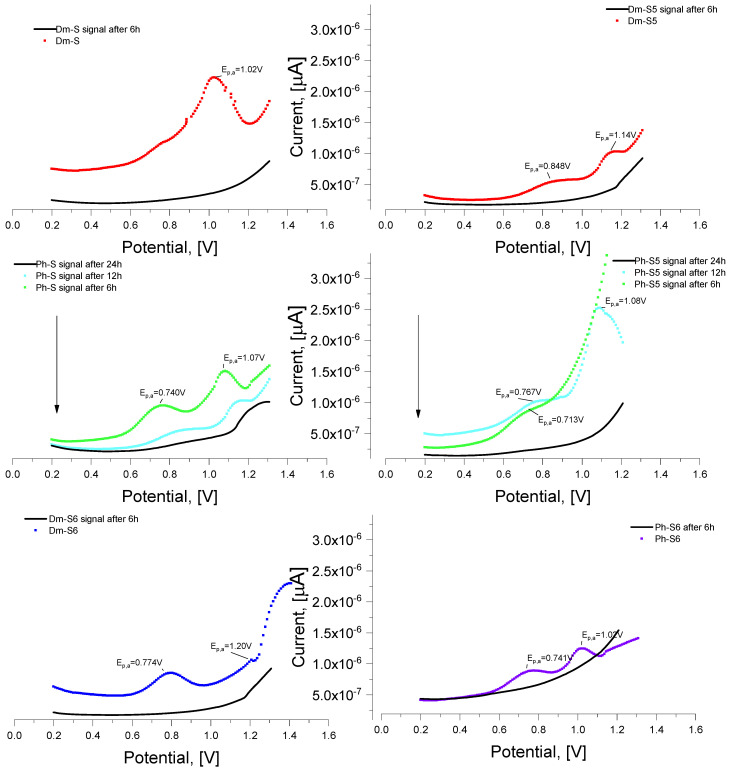
DPP signals of the studied compounds at 40 °C and different incubation times.

In order to determine the stability of the molecular sequence in an environment close to the physiological one at different temperatures from 37 to 40 °C, voltamperometric studies were carried out, showing structural changes at minimal deviations from the baseline conditions under which the signal is registered. The electrochemical oxidation of peptides is mainly due to electroactive amino acid residues from the presented compounds such as tyrosine and tryptophan [28]. In DP voltamperometry at GC electrode and physiological pH, the oxidation potentials of free amino acids are E_p,a_~+0.6 V for tyrosine [29,30] and tryptophan [31]. Electroactive amino acid residues of the molecule can be more easily oxidized when present on the outside of the peptide or are not spatially impeded, thus making contact with the electrode surface easier [32]. As shown by the voltamperograms for all freshly prepared solutions of the peptides at 0 h incubation, two successive oxidation peaks can be distinguished for each peptide, as shown in Figure 7. The first anodic charge transfer reaction corresponds to the oxidation of tyrosine at E_p,a_~0.7 V, and the second more positive peak E_p,a_~1.00 V is more likely to be due to tryptophan as it is also noticeable in pure spinorphin. The anodic signals correspond to those in the literature for peptide sequences containing the electroactive tyrosine and tryptophan [30,32]. From Figure 7, it can be seen that the different surroundings around the active sequences affect the electron exchange in the electrode reaction, reflecting on the graph as a variation in the current intensity of the peaks. After incubation of the peptide compounds at 37 °C for a period of 6, 12, and 24 h, no change in the intensity of the received signals was noticed. With an increasing temperature of 40 °C, however, it was seen that the compounds hydrolyze and the signals of Dm-S, Dm-S5, Dm-S6, and Ph-S6 completely disappear after 6h of incubation (Figure 8). Among the most stable structures are compounds Ph-S and Ph-S5, which can be said to undergo partial hydrolysis (decreasing signal intensity) at higher temperatures (Figure 8) at up to 12 h of incubation. The decrease in the peak oxidation current of these compounds continued until 24 h incubation, as shown in Figure 8, when no signals were recorded. M. Vestergaard et al. proved that in such cases, the molecules of peptides were completely converted into highly ordered aggregates and/or fibrils with electroactive amino acid residues buried inside, which could not reach the electrode surface [32,33].

### 2.4. Results for Anticonvulsant Activity

The six spinorphin peptides (Dm-S, Dm-S5, and Dm-S6) and (Ph-S, Ph-S5, and Ph-S6) were tested in three seizure tests with different mechanisms of action according to the guidelines of the Antiepileptic Drug Development Program (ADD) of the National Institutes of Health (USA) [34]. The neurotoxicity of newly synthesized peptide analogues was also assessed using the motor coordination test (rotarod test). None of the compounds tested showed motor impairment, suggesting the absence of sedative side effects.

Regarding the 6-Hz test, the newly synthesized spinorphin analogues (Dm-S, Dm-S5, and Dm-S6) were inactive against psychomotor seizures unlike their positive control S (Table 2). Among the spinorphin analogues with phenyl residue, the Ph-S and the Ph-S6 showed an efficacy (67% protection) in the 6-Hz test (*p* ≤ 0.05 vs. control) comparable to that of the referent peptide S (Table 3).

Regarding the MES test, in contrast to the positive control S, which was inactive against the generalized seizures, the hexapeptide spinorphin analogue Dm-S6 showed an anticonvulsant effect and 0% mortality at the highest dose of 2.4 µg (67% protection) (*p* ≤ 0.05 vs. control) (Table 4). Both the Ph-S5 and the Ph-S6 were ineffective in suppressing tonic hind limb extension with concomitant low mortality of 17% at the 2.4 µg dose (67% protection) (*p* ≤ 0.05 vs. control) (Table 5).

**Figure 9:**
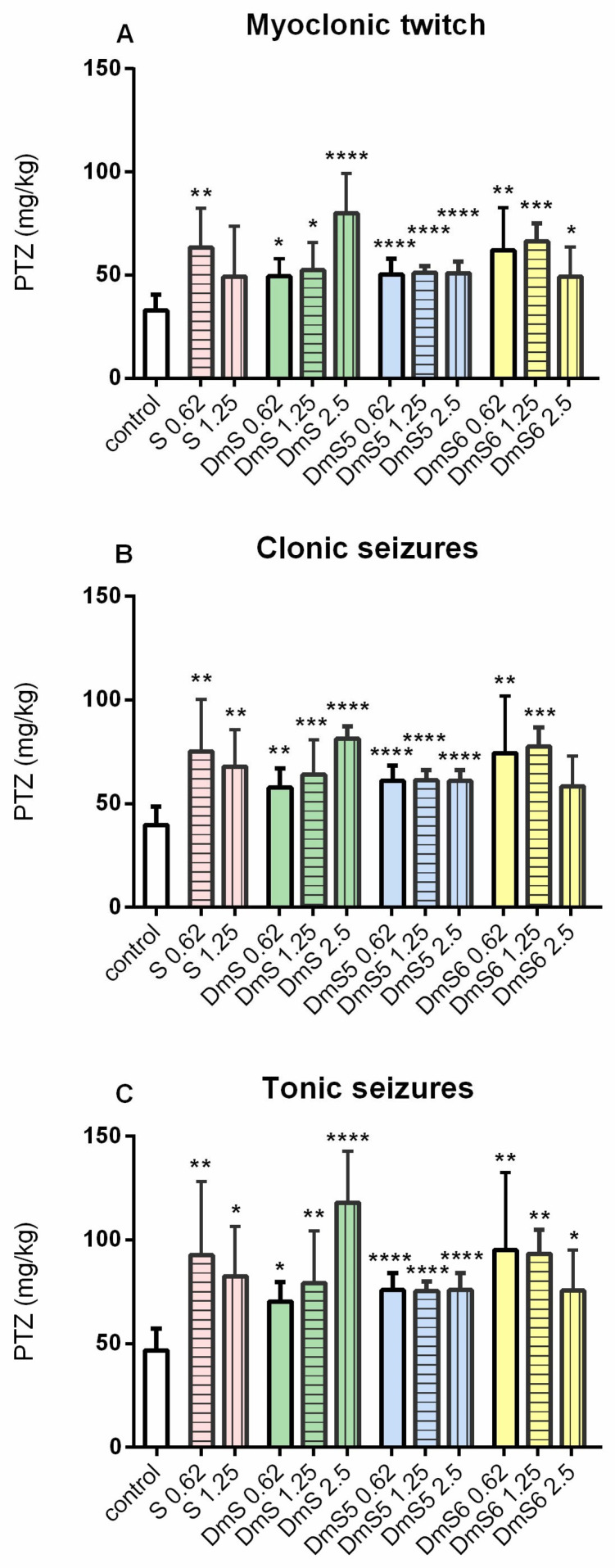
Effect of Dm-S, Dm-S5, and Dm-S6 compounds on the threshold for myoclonic (**A**), clonic (**B**), and tonic (**C**) seizures induced by intravenous pentylenetetrazol (ivPTZ) in mice. Each bar represents the mean (mg/kg PTZ) + S.E.M. Statistical analyses were performed using Student’s *t* test. * *p* ≤ 0.05, ** *p* < 0.01, *** *p* < 0.001 and **** *p* < 0.0001 respectively, compared to control group.

**Figure 10:**
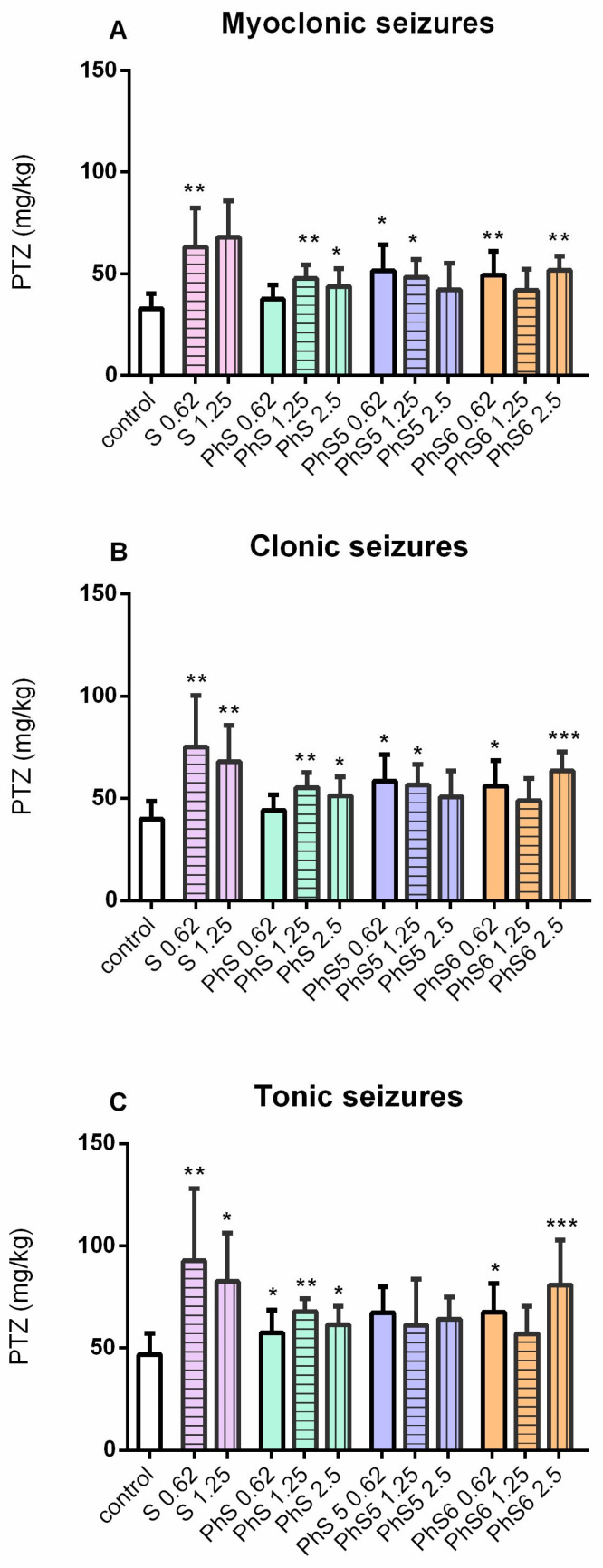
Effect of Ph-S, Ph-S5, and Ph-S6 compounds on the threshold for myoclonic (**A**), clonic (**B**), and tonic (**C**) seizures induced by intravenous pentylenetetrazol (ivPTZ) in mice. Each bar represents the mean (mg/kg PTZ) + S.E (standard error). Statistical analyses were performed using Student’s *t* test. * *p* ≤ 0.05, ** *p* < 0.01, and *** *p* < 0.001, respectively, compared to control group.

Regarding the ivPTZ test, the positive spinorphin control S showed anticonvulsant activity at doses of 0.62 μg and 1.25 μg against myoclonic twitching, clonic seizures, and forelimb tonic seizures compared to the vehicle-infused control group (*p* < 0.05; *p* < 0.01) (Figure 9A–C). In addition, the three Dm peptide analogues (Dm-S, Dm-S5, and Dm-S6) and the three phenyl spinorphin analogues (Ph-S, Ph-S5, and Ph-S6) dose-dependently increased the threshold for the three seizure phases compared to the control (*p* < 0.05) and showed anticonvulsant activity comparable to that of the positive control S (Figure 10A–C).

**Figure 11:**
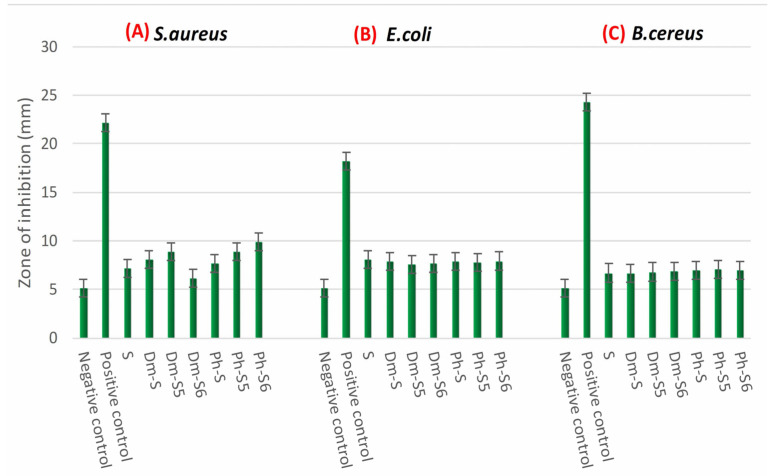
Graphic of the diameter of the inhibitory zone of peptides against (**A**) *S. aureus* bacteria, (**B**) *E. coli* bacteria, and (**C**) *B. cereus* bacteria. Each bar represents the mean + S.E (standard error); negative control: 10%DMSO; positive control: 10 g of chloramphenicol on the paper disc.

**Figure 12:**
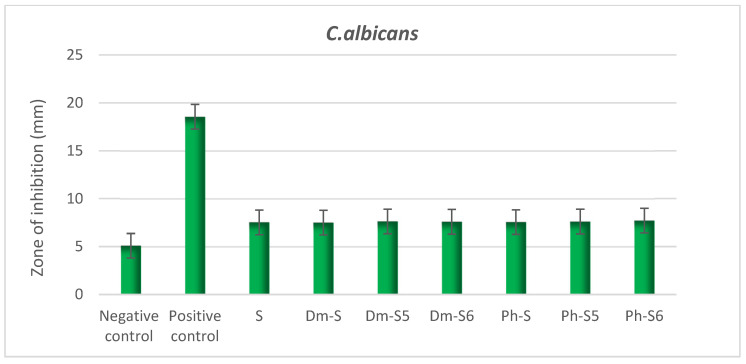
Diameter of the inhibitory zone of peptides against the fungus *Candida albicans*. Each bar represents the mean + S.E (standard error); negative control: 10% DMSO; positive control: nystatin (0.5 g) on the paper disc.

**Figure 13:**
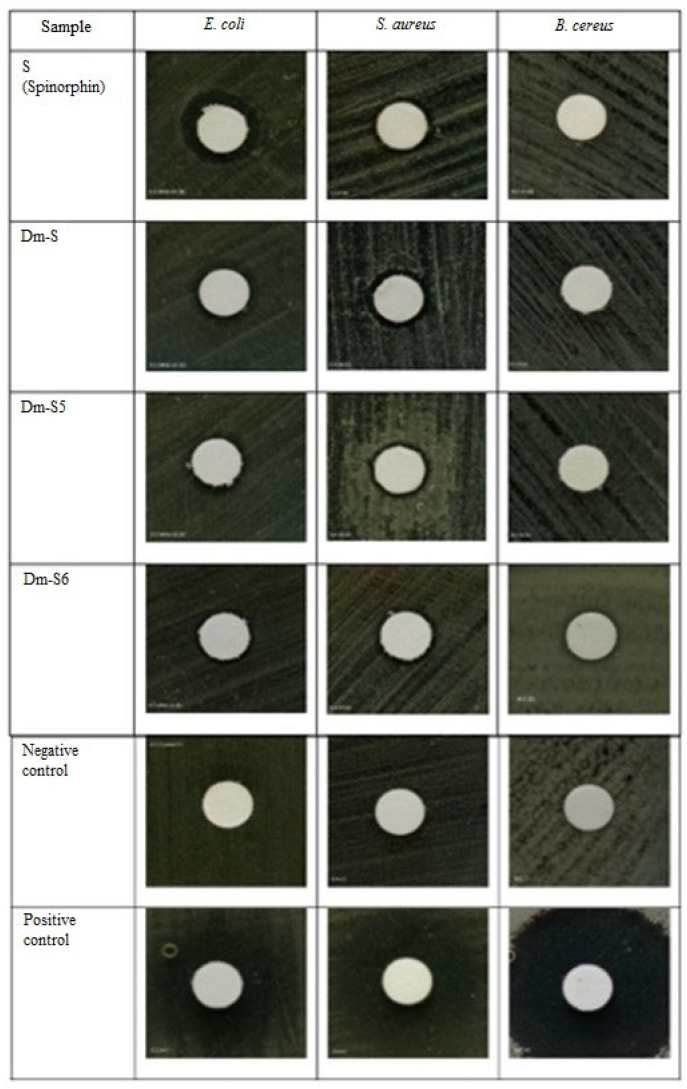
Inhibitory activity of Dm-peptides against test bacteria; negative control: 10% DMSO; positive control: 10 g of chloramphenicol on the paper disc.

Bioactive peptides are protein fragments that have different functions after being released from the original protein and can have significant pharmacological effects. Bioactive peptides can act as antihypertensive, antioxidant, antimicrobial, opioid, mineral-binding, antithrombotic, or immunomodulatory agents based on amino acid composition, size, sequence, and physicochemical properties [35,36]. Peptides are classified as cationic or anionic based on their charge. Cationic peptides kill microbes by interacting with the anionic components of the target cell membrane; most antimicrobe peptides (AMPs) are cationic and target the negatively charged cell wall or plasma membrane. However, anionic peptides use different mechanisms, including translocation across the membrane and pore formation, to cause cell death and loss of vital components [37]. The results obtained by us for the antibacterial activity of the studied peptide derivatives show that synthetic peptide analogues of spinorphin possess antimicrobial activity against bacteria and fungi (Figure 11 and Figure 12). Based on the statistical analysis performed with the subsequent Duncan test with a confidence level of 95% (a = 0.05), the test for antibacterial activity of an analog peptide did not show that statistically significant differences were found for each test of the test bacteria *E. coli*, *S. aureus*, and *B. cereus*, nor as antifungal tests carried out in the group *Candida albicans*. The results of testing the antibacterial activity of peptides using the diffusion method can be seen in Figure 13. Based on Figure 11, the average result of the inhibitory zone diameter for each treatment was higher than the negative control and lower when compared to the positive control (10 g of chloramphenicol on the paper disc). Dm-S5 peptides showed the highest average results when compared to other peptides. Peptide Ph-S6 has the highest ability to inhibit *S. aureus* bacteria compared to other peptides. The results of testing the antibacterial activity of peptides against *E. coli* test bacteria revealed that spinorphin outperformed peptide analogues. This demonstrates spinorphin’s potency in inhibiting the growth of *E. coli* bacteria. According to the graph of the average inhibitory zone of peptides against *B. cereus* bacteria, Dm-S has the lowest average. This demonstrates that, when compared to other peptides, Dm-S6 peptides have a high ability to inhibit the growth of *B. cereus* bacteria. The results of testing antifungal activity with the candida albicans test fungus showed that the Dm-S6 peptide has the highest antifungal activity when compared to other peptides. However, some references point out that the antimicrobial activity test is a qualitative test. However, the presence of antimicrobial activity is sufficient to prove that the tested peptides contain active groups, thanks to which their antimicrobial properties are manifested.

### 2.5. Antioxidant Activity Test

**Figure 14:**
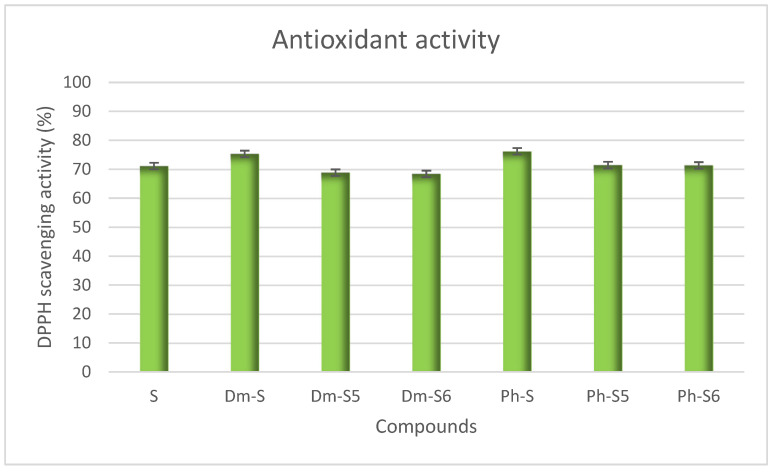
Antioxidant activity of peptides. Each bar represents the mean + S.E.

Antioxidant compounds can protect cells from oxidative stress, which can damage macromolecules such as proteins, lipids, and DNA [38]. Individual amino acid residues influence the antioxidant activity of the peptide series. Tyr, Met, Lys, Cys, and His are amino acid residues found in the structure of antioxidant peptides. In addition to their role in the peptide sequences, amino acid residues at the N and C ends are thought to be more important in determining antioxidant activity. With the advent of more hydrophobic amino acids, the last three amino acids in the C-terminal play an important role. The hydrophobic properties of peptides increase their lipid solubility and allow them to interact with hydrophobic and lipid radicals. Gly, Ala, Val, Leu, Ser, and Pro are found in the N-terminal sequence of some antioxidant peptides. Aromatic amino acids (Tyr, His, Trp, and Phe) can donate electrons to radicals, making them important in peptide radical trapping activity [39]. Previous research has also found that Tyr and Trp play important roles in peptide antioxidant activity. Due to the presence of indole groups, tryptophan can chelate metal ions, purify hydroxyl radicals, and inhibit lipid oxidation. The results of testing antioxidant activity on peptide analogues using the same statistical test showed seemingly different results (Figure 14). It can be concluded that the compounds that do not contain Ac5c and Ac6c have a higher antioxidant ability. The location of the two substituents between the antioxidant-active tyrosine and tryptophan clearly affects their ability to neutralize free radicals without the antioxidant activity of the molecule (Figure 14).

## 3. Materials and Methods

### 3.1. Synthesis of Peptide Analogues

#### General Procedure for the Peptide Synthesis of Compounds (Dm-S, Dm-S5, Dm-S6, Ph-S, Ph-S5, and Ph-S6)

All N-modified peptide analogs of spinorphin were synthesized manually using the solid-phase method using Fmoc chemistry, and the procedure and reagents used are detailed in [13]. Peptides were produced using Fmoc-Rink-Amide MBHA resin (loading 0.71 mmol/g resin; crosslinking 1% DVB; 100–200 mesh). Peptide chains were lengthened in successive cycles of deprotection and coupling. The coupling reactions were performed using amino acid/TBTU/HOBt(hydroxybenzotriazole)/DIPEA(N,N-diisopropylethylamine)/resin at a molar ratio of 3/2.9/3/6/1, in a 1:1 combination of DMF (N,N-dimethylformamide)/DCM (dichloromethane) [18]. The peptides were obtained as white powders with a purity of >96% as determined by analytical HPLC. The structures were confirmed by high-resolution electrospray mass spectrometry. Peptide purity was monitored using reversed-phase high-performance liquid chromatography (RP-HPLC), column: SymmetryShieldTM RP-18, 3.5 μ, (50 × 4.6 mm), flow: 1 mL/min, H_2_O (0.1% TFA)/CH_3_CN (0.1% TFA), gradient 0→100% (45 min) and 100% (5 min). The crude peptides were purified by semi-preparative HPLC on column XBridgeTM Prep C18 10 μm (10 × 250 mm), flow: 5 mL/min, H_2_O (0.1% TFA)/CH_3_CN (0.1% TFA), gradient 20→100% (50 min). All analytical data are summarized in Table 1.

### 3.2. Physicochemical Characterization

The chemicals utilized were of high purity, specifically analytical grade, and the solutions were prepared using deionized water. The spinorphin peptide molecules were dissolved in water: methanol = 2:1 to create solutions with the following concentrations: The concentration of S is 1.584 × 10^−3^ mol L^−1^, Dm-S is 1.353 × 10^−3^ mol L^−1^, Dm-S5 is 1.335 × 10^−3^ mol L^−1^, and Dm-S6 is 1.387 × 10^−3^ mol L^−1^. The concentrations of Ph-S, Ph-S5, and Ph-S6 are as follow: 1.230 × 10^−3^ mol L^−1^, 1.469 × 10^−3^ mol L^−1^, and 1.160 × 10^−3^ mol L^−1^. The UV-VIS absorption spectra of all compounds were measured in the wavelength range of 200–600 nm using a UV-1280 UV VIS spectrophotometer (Shimadzu). The spectrophotometer was operated in double-beam mode, and quartz cuvettes with an optical path length of 1.0 cm were used.

#### 3.2.1. Determination of Partition Coefficient

The spinorphin derivatives’ partition coefficient was measured in a solution consisting of 1-octanol and phosphate buffer (NaH_2_PO_4_/Na_2_HPO_4_, pH 7.41 ± 0.01) using the flask-shaking method. In order to achieve mutual saturation in both the 4 mL buffer and 4 mL 1-octanol phases, a quantity of the test compounds (2.00 mL standard water: methanol solutions) was introduced into an 8 mL distribution system that had been vigorously stirred for 12 h at room temperature (25 °C). Following the separation process, the emission of the solutions from both phases were quantified, and the concentration of the dissolved analyte was estimated using the standard additions technique. Peptide stock solutions were utilized as reference samples to calculate the coefficients for the analytical function. The partition coefficient was determined using the following equation: logP = log(C^oct/buf^/C^buf/oct^). This equation represents the relationship between P, C^oct/buf^, and C^buf/oct^. C^oct/buf^ and C^buf/oct^ refer to the molar concentrations of the solute in the mutually saturated phases of 1-octanol and buffer.

#### 3.2.2. Voltamperometric Analysis

The compounds were electrochemically characterized using a computer-controlled electrochemical system, specifically a Metrohm 797 VA trace analyzer with a 797 VA stand. The system operated in differential pulse mode. The electrochemical cell consisted of a working glass carbon (GC) electrode, a reference electrode (Ag/AgCl, KCl (3.0 mol × L^−1^), and a carbon auxiliary electrode. Differential pulse (DPP) voltammetry experimental conditions were a pulse amplitude of 50 mV and a scan rate of 5 mV s^−1^.

The signals were measured in a phosphate buffer solution with a concentration of 0.100 mol × L^−1^ and a pH of 7.41 ± 0.01. The measurement was conducted in a high-purity nitrogen environment at room temperature, which was maintained at 25 ± 1 °C. The analytical technique consisted of the following steps: A phosphate buffer solution with a volume of 7.00 mL was added to a glass voltammetric cell with a capacity of 50 cm^3^. The voltammetric curve was then recorded in order to identify any interference signals. Successive aliquots of the standard peptide solutions, ranging from 50 to 400 µL, were added to the electrolyte solution that had been previously degassed with high-purity nitrogen for a duration of 10 min. The data are reported as the average of three separate measurements. A possible deviation of the maximum when making several recordings of the signal would mean that during the time period of recording the signal, there are unwanted processes or a possible malfunction of the equipment (uncalibrated equipment or otherwise). In our case, when writing out the voltammograms, this was not noticed.

#### 3.2.3. Hydrolytic Activity Testing

The hydrolytic stability of all new compounds was tested in the aqueous medium at physiological pH values (0.1 mol × L^−1^ phosphatic buffer solution with pH = 7.41 ± 0.01). A 5 μM aqueous solution of the compounds was prepared from a 5 mM stock solution in phosphate buffer and incubated at 37 °C and 40 °C for intervals of 6, 12, and 24 h; samples taken at different times were analyzed voltamperometrically on a glass carbon electrode in DPP mode.

#### 3.2.4. pK and pI Determination

Potentiometric titrations were performed to determine acid–base constants at room temperature using a potentiometric cell with a combined glass electrode and OMINS titration system (Metrohm). An OMNIS Digital Burette with an accuracy of ±0.01 mL was used to precisely add the titrant portions to the titrated aliquot (12 µmol) of the stock solution of the peptide compounds. The potentiometric titration results were processed mathematically to construct the titration curves and calculate the acid–base constants according to Hessenbach’s equation [16]. Isoelectric points correspond to pH points where the analyte and titrant interact in equimolar amounts (pH at the equivalence point).

#### 3.2.5. Spectral Characterizations

The ATR-IR spectra for peptide structures were recorded on a Thermo Nicolet iS50 infrared spectrometer in the mid-infrared region from 4000–600 cm^−1^ with a diamond crystal ATR accessory at a resolution of 2 cm^−1^ and 64 scans. The absorption and emission/excitation spectra of the compounds under investigation were measured using a Varian-Cary spectrophotometer equipped with synthetic quartz glass cells with a path length of 1 cm in the UV-Vis region. The spectra were also recorded using a Cary Eclipse spectrofluorometer (Agilent, Santa Clara, CA, USA) in the range of 200–900 nm, with a resolution of 0.5 nm. The spectrofluorometer employed double-grating monochromators for excitation and emissions, respectively. The CD spectra were determined using a Jasco J-1500 CD spectrometer, which has a spectral range of 160–800 nm and a resolution of 1 nm. The spectrum measurements, which were influenced by temperature, were conducted using an external circulating thermostat and a thermocouple that made direct contact with the sample. The spectra were measured within a temperature range of 25–35 °C, with increments of 3 °C. The nitrogen flow rate was consistently maintained at 4–5 L per minute. The morphology of newly synthesized peptides was analyzed using a JSM-IT200 Scanning Electron Microscope.

### 3.3. Study of Bioactive Properties

#### 3.3.1. Pharmacology: In Vivo Experiments

- Animals:

Male ICR mice (25–30 g) were obtained from the vivarium of the Institute of Neurobiology, BAS. They were acclimatized to standard conditions for one week prior to experiments as follows: light/dark (12/12) regime, 22 ± 2 °C room temperature, 40% humidity, and access to food and water ad libitum. The procedures were performed in accordance with the European Communities Council Directive 2010/63/EU and were approved by the Bulgarian Food Safety Agency (Licence No: 354).

- Drugs and dosage

The spinorphin peptide analogues (S, Dm-S, Dm-S5, and Dm-S6 or PhS, PhS5, and PhS6) were administered intracerebroventricularly (i.c.v.) (10 μL/ventricle) at doses of 0.6, 1.2, and 2.4 μg, respectively, using a 28-gauge stainless-steel needle attached to a 50-μL Hamilton^®^ syringe as previously described [9]. The matched group (control) was treated with a vehicle. The seizure test was performed 10 min after the i.c.v. infusion.

Intravenous Pentylenetetrazole Seizure (ivPTZ) Test The convulsant pentylenetetrazole (PTZ) (1%), dissolved in saline, was infused (0.005 mL/s) into a tail vein as previously performed [40]. The activity to raise the threshold of ivPTZ-induced myoclonic, clonic, and tonic seizures and concentrations, quantity of infusion (mL), and mouse body weight were recorded.

- Seizure tests

6-Hz test. Psychomotor seizures were induced by corneal electrodes (constant current shock generator, 32 mA, 6 Hz, 3 s) as previously described [9]. The return to a normal position, 10 s after stimulation, was used as a criterion for protection against psychomotor seizures.

- Maximal electroshock Test (MES test).

The generalized seizures were induced through a stronger stimulus of 50 mA, 60 Hz, 0.2 s, applied via the same corneal electrodes, in each mouse tested. The suppression of tonic hind limb extension, replaced by a clonic seizure, was accepted as a valid criterion for the anticonvulsant potency of the drug.

- Rota-rod test

The rota-rod apparatus was used to assess potential drug neurotoxicity. Mice were gently placed on a rotating rod (3.2 cm in diameter, at a speed of 10 rpm) and the ability to maintain motor coordination on the rod for at least one minute was assessed out of three possible sessions.

- Statistical analysis

Fisher’s exact was used for the analysis of data (Sigmastat, version SigmaStat^®^ 11.0). Statistical significance was accepted at *p* ≤ 0.05.

#### 3.3.2. Microbiological Analyses: Antibacterial Activity, Antifungal, and Antioxidant Test

- Antibacterial Activity Test

Antibacterial activity is carried out with reference to the slightly modified method of Murray et al. [41]. The bacteria used were *Staphylococcus aureus*, *Escherichia coli*, and *Bacillus cereus.* To test the antibacterial activity of 6 peptides, it is carried out as follows: taking 100 μL of spectrum bacteria (10 CFU/mL bacteria) spread on the nutrient agar medium (NA). Furthermore, a paper disc (6 mm in diameter) was placed and then 20 μL of extract with a concentration of 50 mg/mL was dripped onto it. As a positive control, we used 10 g of chloramphenicol on the paper disc. DMSO 10% is used as a negative control according to peptide solvents. The treatment was repeated three times and then incubated for 24 h at a temperature of 37 °C. After that, an inhibitory power test measurement was carried out by measuring the diameter of the clear zone formed [42].

- Antifungal Activity

Antifungal activity in peptide samples was carried out by diffusion in order to look at the diameter of the inhibition zone contained around the paper disc. Testing was carried out on the fungus Candida albicans. The concentration of the extract tested was 50 mg/mL. The culture of each test fungus was aseptically taken from the oblique agar using a dosing needle and rejuvenated in a liquid medium. In each medium, there is a spore density of 10 CFU/mL. Furthermore, the sabouraud agar (SA) medium was prepared in a petri dish and each culture was scratched on top of the agar, then placed on a paper disc (disc paper), and finally, a peptide sample of 20 µL was placed on the paper disc. It was further incubated for 24 h and we measured the zone of the formed obstacle. A 10% solution of DMSO was used as a negative control, and nystatin (0.5g) on the paper disc was used as the positive control.

- Antioxidant Activity

The determination of the antioxidant activity of peptides was carried out by inserting 77 μL of the peptide extract into a test tube, then adding 3 mL of the DPPH (2,2-diphenyl-1-picrylhydrazyl) solution, homogenizing, and incubating for 30 min in a dark room. The measurement of antioxidant activity was performed using a spectrophotometer at a wavelength of 517 nm. The determination of activity for the standard solution was conducted by first diluting 0.025 BHT (Butylated Hydroxytoluene) with 1 mL of methanol. Then, 3 mL of DPPH was added to 77 μL of the BHT solution in a test tube, homogenized, and incubated for 30 min in a dark room, and a measurement of absorbance of the solution at the same wavelength (517 nm) was taken. All treatments were repeated three times. For the blank, only 77 μL of methanol plus 3 mL of DPPH was used, then homogenized and incubated for 30 min in a dark room. According to S.M Khamsah et al. (2006) [43], the free radical scavenging activity of the sample was calculated using the formula:

$$DPPH\;radical\;concentration\;(\%)\;=\frac{A\left(Control\right)-A(Sample)}{A\;(Control)}\;\times \;100$$

where A_Control_ is the absorbance value of the control reaction and A_sample_ is the absorbance value with the presence of the tested extracts in the sample.

## 4. Conclusions

Some novel 5,5′-dimethyl- and 5,5′-diphenylhydantoin-conjugated spinorphin derivatives were synthesized by a solid-phase peptide synthesis (SPPS)-Fmoc (9-fluorenylmethoxy-carbonyl) strategy and fully characterized using various methods and techniques. The structure of the new spinorphin analogs was significantly altered due to the rearrangement of amino acid residues and modification of the molecule with hydantoin rings at the N-terminus and with Ac5c and Ac6c in the fifth position in the peptide chain. In terms of the behavior of newly synthesized compounds in physiological media, it should be highlighted that the conjugated component of the molecule has no significant impact on their physiochemical properties. All studied peptides appear to be weak protolytes, forming ions at physiological pH and being stable under these conditions for biological activities. The spectral and electrochemical profiles of the compounds were determined to demonstrate their molecular stability under various environmental conditions. Fluorescence analysis revealed logP values that characterized the compounds as lipophilic rather than hydrophilic. It indicates that compounds accumulate in adipose tissue, pass through the lipid layers of biological membranes, and so have the potential for bioaccumulation in organisms. The compounds demonstrated good structural stability when examined voltammetrically, as they may reach the required centers for the manifestation of biological activity without hydrolysis processes at a body temperature of 37 °C and physiological pH. After 6 h of incubation, increasing the temperature to 40 °C revealed structural alterations associated with potential hydrolysis of the Dm-S, Dm-S5, Dm-S6, and Ph-S6. Compounds Ph-S and Ph-S5 have the most stable structures. They undergo partial hydrolysis (decreased signal strength) at higher temperatures with up to 12 h of incubation and an entire structural change until 24 h of incubation when no voltamperometric signals were recorded. The analysis of the microstructure–bioactivity attributes showed that the spinorphin analogue peptides that were synthesized have the ability to function as both antibacterial and antioxidant substances. Further research on them may lead to the development of alternative drugs or applications in other fields. The two spinorphin analog peptides with phenyl and the Dm group showed activity against the three seizure phases of the ivPTZ test. This finding suggests that these residues do not play a crucial role in the structure of spinorphin compounds and determine the potency of raising the seizure threshold. Similarly, the two spinorphin peptides with six amino acid residues (Dm-S6 and Ph-S6) showed similar potency against the seizure spreading (the MES test) suggesting that the 5,5′-dimethyl- and 5,5′-diphenylhydantoin residues are not responsible for enhancing the anticonvulsant activity of spinorphin peptides against tonic seizures. On the other hand, spinorphin analogs with diphenyl- but not dimethyl- residues were active against the drug-resistant epilepsy test (6-Hz test).

## Figures and Tables

**Table 1:** Analytical data of synthetic spinorphin peptides containing hydantoin moiety.

№	Peptide	^a^ [α]_546_^20 (o)^	Molecular Formula	^b^ tR, min	^c^ [MH] + Calculated	^c^ [MH] + Observed	LogP	pKa_1_	pKa_2_	pI_1_; pI_2_
**S**	H-Leu-Val-Val-Tyr-Pro-Trp-Thr-NH_2_	−74	C_45_H_65_N_9_O_9_	20.04	876.0525	876.4970	0.421	3.15	6.78	4.91; 8.31
**Dm-S**	Dm-Leu-Val-Val-Tyr-Pro-Trp-Thr-NH_2_	−70	C_52_H_73_N_11_O_12_	26.88	1044.2025	1044.5501	1.16	3.18	7.16	5.07; 8.35
**Dm-S5**	Dm-Leu-Val-Val-Tyr-Ac5c-Trp-Thr-NH_2_	−20	C_53_H_75_N_11_O_12_	33.76	1058.2291	1058.5654	1.08	3.19	-	4.91; 8.30
**Dm-S6**	Dm-Leu-Val-Val-Tyr-Ac6c-Trp-Thr-NH_2_	−32	C_54_H_77_N_11_O_12_	36.63	1072.2557	1072.5814	0.934	3.31	7.23	5.04; 8.31
**Ph-S**	Ph-Leu-Val-Val-Tyr-Pro-Trp-Thr-NH_2_	−68	C_62_H_77_N_11_O_12_	38.64	1168.3413	1168.5799	1.01	3.20	-	5.06
**Ph-S5**	Ph-Leu-Val-Val-Tyr-Ac5c-Trp-Thr-NH_2_	−72	C_63_H_79_N_11_O_12_	45.72	1182.3679	1182.5957	1.24	3.22	-	5.09
**Ph-S6**	Ph-Leu-Val-Val-Tyr-Ac6c-Trp-Thr-NH_2_	−20	C_64_H_81_N_11_O_12_	45.00	1196.3944	1196.6118	1.35	3.21	6.84	5.06; 8.06

^a^ Optical rotation in methanol (c = 0.01) at 20 °C; ^b^ t_R_ is the retention time determined by RP-HPLC; ^c^ the mass ion (MH^+^) was obtained by electrospray ionization mass spectrometry (ESI-MS) (Figures S1–S6).

**Table 2:** The activity of S, Dm-S, Dm-S5, and Dm-S6 in the 6-Hz test in mice.

Group	Dose µg/10 µL	No. of Animals Protected/No. of Animals Tested	% Protection
**control**	0	0/8	0%
**S**	0.62	3/6	50%
	1.25	3/6	50%
	2.5	4/6	67% *
**Dm-S**	0.62	2/6	33%
	1.25	2/6	33%
	2.5	2/6	33%
**Dm-S5**	0.62	2/5	40%
	1.25	2/5	40%
	2.5	3/6	50%
**Dm-S6**	0.62	1/6	17%
	1.25	3/6	50%
	2.5	3/6	50%

Statistical analyses were performed using Fisher’s exact test. * *p* ≤ 0.05 compared to control.

**Table 3:** The activity of **Ph-S**, **Ph-S5,** and **Ph-S6** in 6-Hz test in mice.

Group	Dose µg/10 µL	No. of Animals Protected/No. of Animals Tested	% Protection
**control**	0	0/8	0%
**Ph-S**	0.62	4/6	33%
	1.25	4/6	67% *
	2.5	2/6	67% *
**Ph-S5**	0.62	1/6	17%
	1.25	3/6	50%
	2.5	3/6	50%
**Ph-S6**	0.62	1/6	17%
	1.25	3/6	50%
	2.5	4/6	67% *

Statistical analyses were performed using Fisher’s exact test. * *p* ≤ 0.05 compared to control.

**Table 4:** The activity of **S**, **Dm-S**, **Dm-S5**, and **Dm-S6** in the MES test in mice.

Group	Dose µg/10 µL	No. of Animals Protected/No. of Animals Tested	% Protection	% Mortality
**control**	0	0/8	0%	30%
**S**	0.62	2/6	40%	0%
	1.25	2/6	40%	30%
	2.5	3/6	50%	50%
**Dm-S**	0.62	0/6	0%	83%
	1.25	1/6	17%	33%
	2.5	2/6	33%	50%
**Dm-S5**	0.62	0/6	0%	33%
	1.25	1/6	17%	33%
	2.5	2/6	33%	33%
**Dm-S6**	0.62	1/6	17%	33%
	1.25	2/6	33%	50%
	2.5	4/6	67% *	0%

Statistical analyses were performed using Fisher’s exact test. * *p* ≤ 0.05 compared to control.

**Table 5:** The activity of Ph-S, Ph-S5, and Ph-S6 in MES test in mice.

Group	Dose µg/10 µL	No. of Animals Protected/No. of Animals Tested	% Protection	% Mortality
**control**	0	0/8	0%	30%
**Ph-S**	0.62	2/6	33%	0%
	1.25	3/6	50%	50%
	2.5	3/6	50%	17%
**Ph-S5**	0.62	2/6	33%	50%
	1.25	3/6	50%	33%
	2.5	4/6	67% *	17%
**Ph-S6**	0.62	2/6	40%	60%
	1.25	3/6	50%	67%
	2.5	4/6	67% *	17%

Statistical analyses were performed using Fisher’s exact test. * *p* ≤ 0.05 compared to control.

## Data Availability

Data are contained within the article and Supplementary Materials.

## Supplementary Materials

The following supporting information can be downloaded at: https://www.mdpi.com/article/10.3390/ph17060770/s1. Figure S1: Mass spectrum of compound Dm-S obtained by electrospray ionization mass spectrometry (ESI-MS); Figure S2. Mass spectrum of compound Dm-S5 obtained by electrospray ionization mass spectrometry (ESI-MS); Figure S3. Mass spectrum of compound Dm-S6 obtained by electrospray ionization mass spectrometry (ESI-MS); Figure S4. Mass spectrum of compound Ph-S obtained by electrospray ionization mass spectrometry (ESI-MS); Figure S5. Mass spectrum of compound Ph-S5 obtained by electrospray ionization mass spectrometry (ESI-MS); Figure S6. Mass spectrum of compound Ph-S6 obtained by electrospray ionization mass spectrometry (ESI-MS).
