# Supplementary material for: Chemical Behavior and Bioactive Properties of Spinorphin Conjugated to 5,5′-Dimethyl- and 5,5′-Diphenylhydantoin Analogs
Source: Pharmaceuticals (Basel). 2024 Jun 12;17(6):770. doi: 10.3390/ph17060770 (PMC11206695; doi:10.3390/ph17060770)
Supplement: Supplementary file 1 [file pharmaceuticals-17-00770-s001.zip › pharmaceuticals-3012977-supplementary/siplementary files.pdf]

Chemical behavior and Bioactive Properties of Spinorphin Conjugated to 5,5'-dimethyl- and 5,5'-diphenylhydantoin Analogs

Stela Georgieva[1], Petar Todorov[2], Jana Tchekalarova[3], S. Subaer[4], Petia Peneva[2], H. Hartati[4], S. Faika[4]

[1]*Department of Analytical Chemistry, University of Chemical Technology and Metallurgy, 1756 Sofia, Bulgaria, e-mail:* st.georgieva@uctm.edu
[2]*Department of Organic Chemistry, University of Chemical Technology and Metallurgy, 1756 Sofia, Bulgaria, e-mail:* pepi_37@abv.bg
[3]*Institute of Neurobiology, Bulgarian Academy of Sciences, 1113 Sofia, Bulgaria. E-mail:* janetchekalarova@gmail.com
[4]*Material Physics Laboratory, Physics Department, Universitas Negeri, Makassar(UNM), Makassar 90223, Indonesia, e-mail:* subaer@unm.ac.id
* Correspondence: st.georgieva@uctm.edu ; Tel.: +359 8163400; S.Georgieva

To confirm the structure of the studied spinorphin structures, their mass spectra were taken (Fig. S1-S6). The mass ion (MH+) was obtained by electrospray ionization mass spectrometry (ESI-MS), as follow:

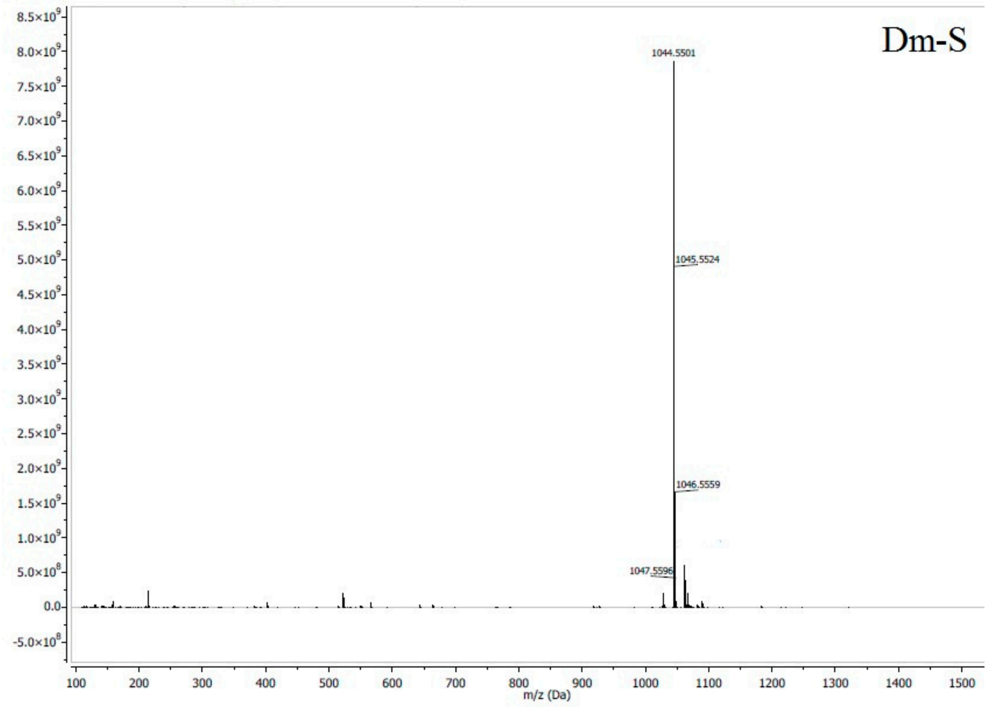

Figure S1. Mass spectrum of compound Dm-S obtained by electrospray ionization mass spectrometry (ESI-MS)

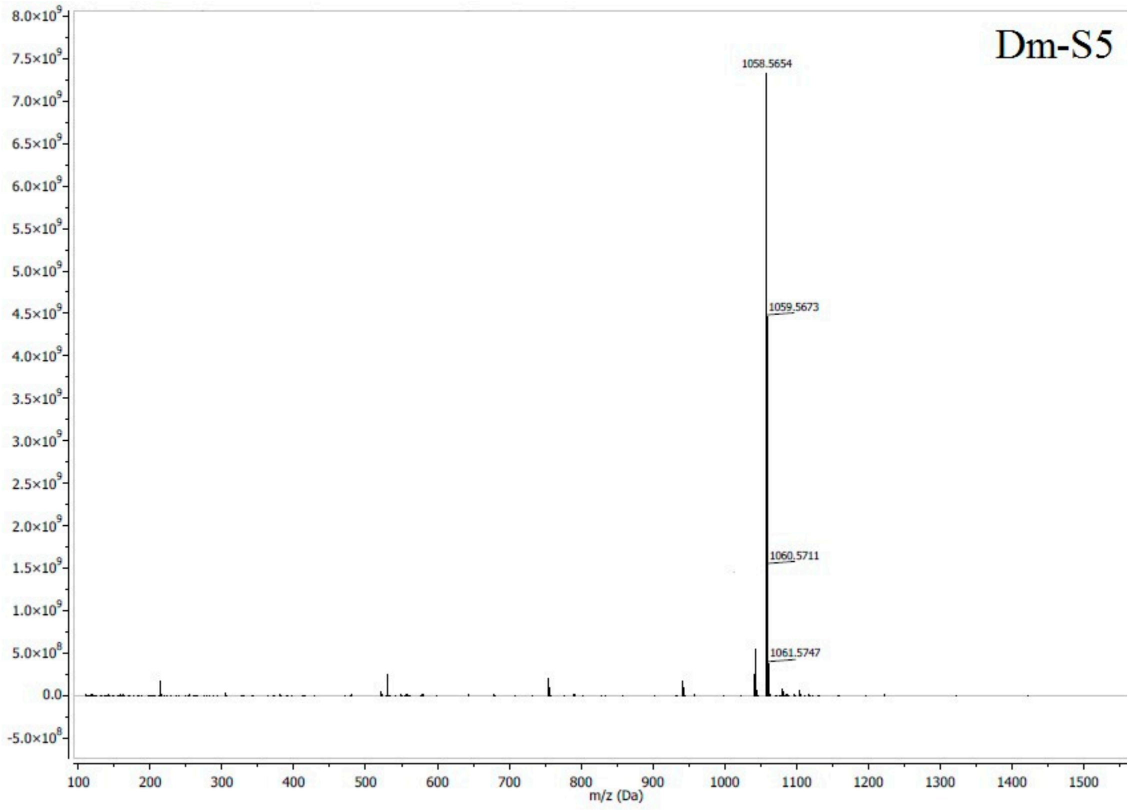

Figure S2. Mass spectrum of compound Dm-S5 obtained by electrospray ionization mass spectrometry (ESI-MS)

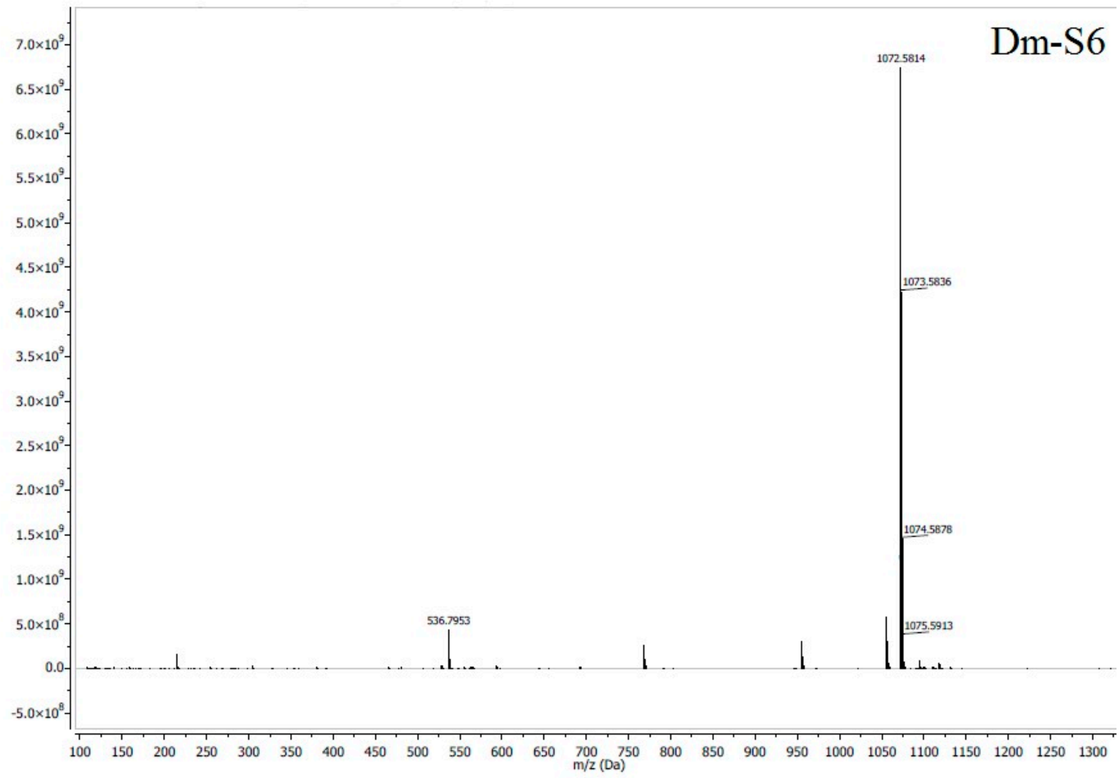

Figure S3. Mass spectrum of compound Dm-S6 obtained by electrospray ionization mass spectrometry (ESI-MS)

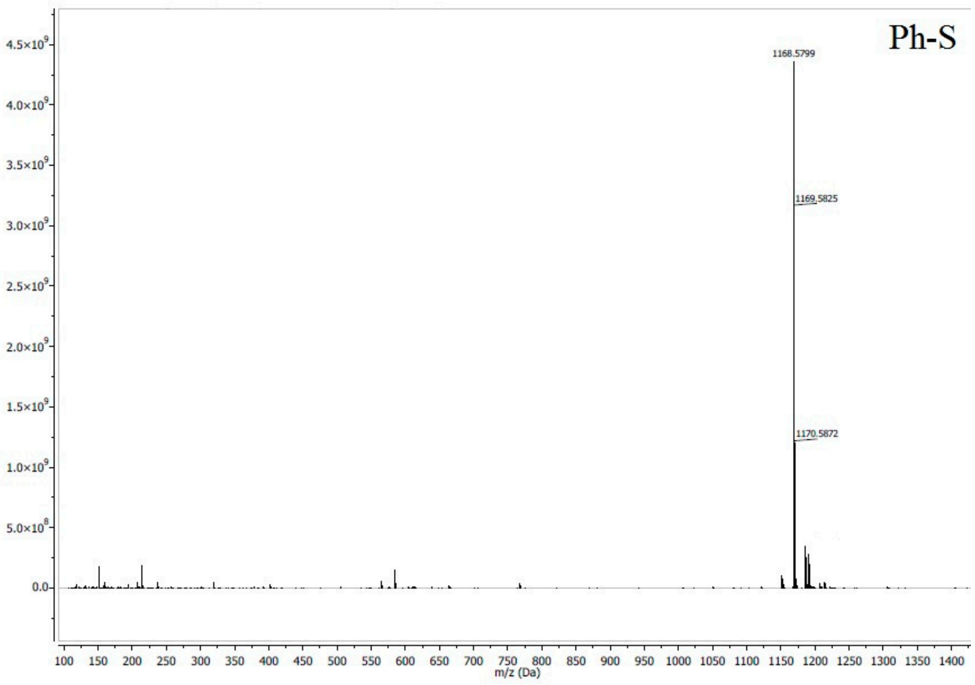

Figure S4. Mass spectrum of compound Ph-S obtained by electrospray ionization mass spectrometry (ESI-MS)

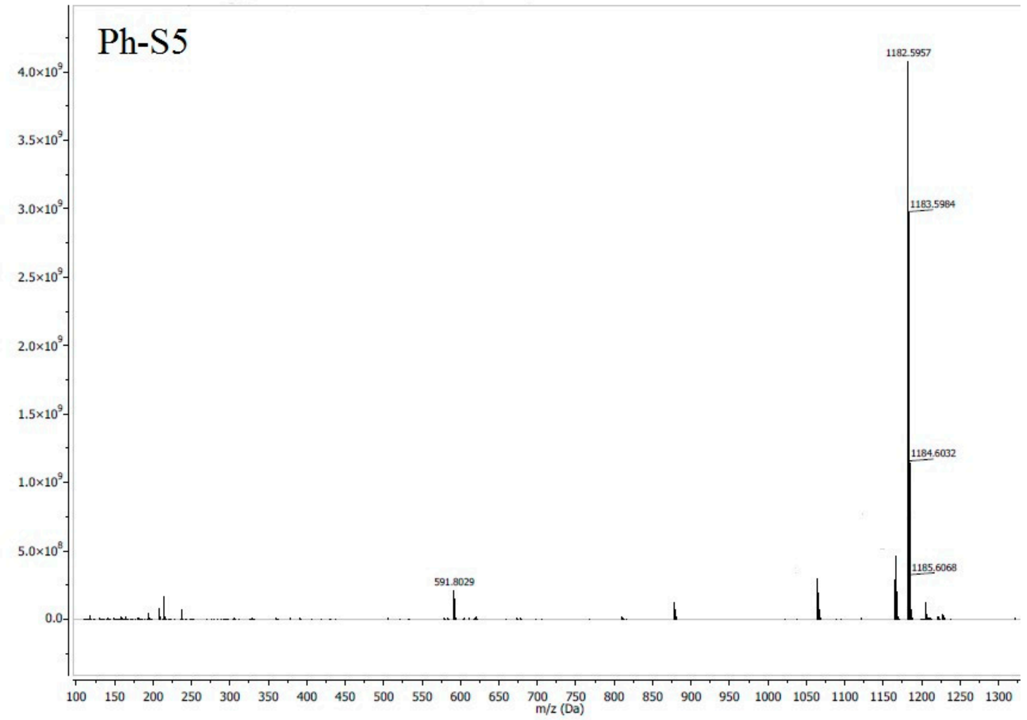

Figure S5. Mass spectrum of compound Ph-S5 obtained by electrospray ionization mass spectrometry (ESI-MS)

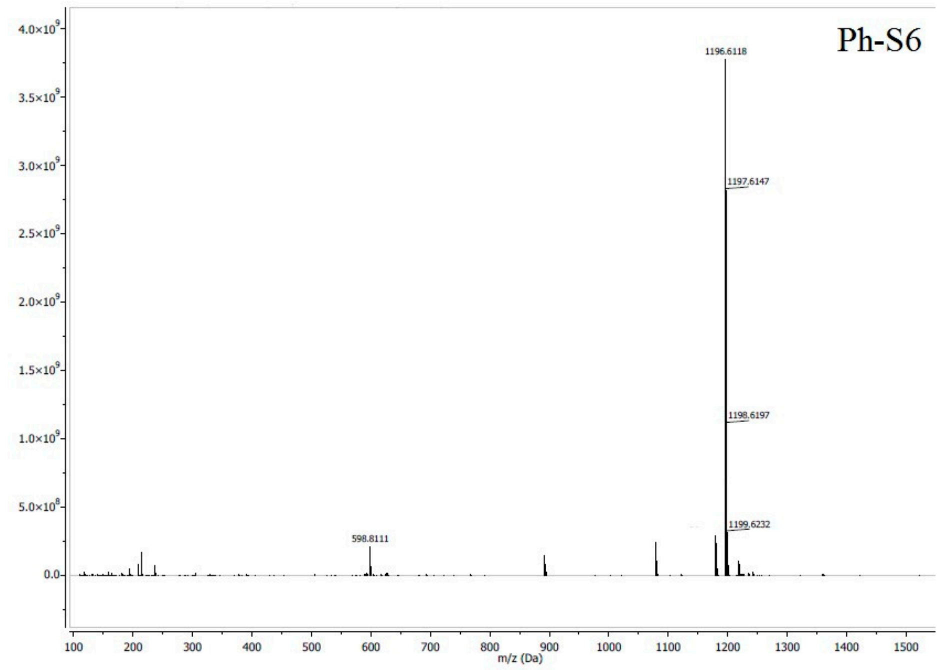

Figure S6. Mass spectrum of compound Ph-S6 obtained by electrospray ionization mass spectrometry (ESI-MS)
